# Antibacterial and Immunomodulatory Potentials of Biosynthesized Ag, Au, Ag-Au Bimetallic Alloy Nanoparticles Using the *Asparagus racemosus* Root Extract

**DOI:** 10.3390/nano10122453

**Published:** 2020-12-08

**Authors:** Musarat Amina, Nawal M. Al Musayeib, Nawal A. Alarfaj, Maha F. El-Tohamy, Gadah A. Al-Hamoud

**Affiliations:** 1 Department of Pharmacognosy, Pharmacy College, King Saud University, Riyadh 11451, Saudi Arabia; mamina@ksu.edu.sa (M.A.); nalmusayeib@ksu.edu.sa (N.M.A.M.); galhamoud@ksu.edu.sa (G.A.A.-H.); 2 Department of Chemistry, College of Science, King Saud University, P.O. Box 22452, Riyadh 11451, Saudi Arabia; moraby@ksu.edu.sa

**Keywords:** green synthesis, bimetallic nanoalloy, *Asparagus racemosus*, antibacterial, immuomodulatory, cytokines

## Abstract

Two noble metals, such as silver and gold alloy nanoparticles, were successfully synthesized by the microwave assisted method in the presence of the *Asparagus racemosus* root extract and were used as an antibacterial and immunomodulatory agent. The nanostuctures of the synthesized nanoparticles were confirmed by various spectroscopic and microscopic techniques. The UV-vis spectrum exhibits a distinct absorption peak at 483 nm for the bimetallic alloy nanoparticles. The microscopic analysis revealed the spherical shaped morphology of the biosynthesized nanoparticles with a particle size of 10–50 nm. The antibacterial potential of the green synthesized single metal (AgNPs and AuNPs) and bimetallic alloy nanoparticles was tested against five bacterial strains. The bimetallic alloy nanoparticles displayed the highest zone of inhibition against *P. aeurgnosia* and *S.aureus* strains when compared to single metal nanoparticles and plant extract. In addition, the inmmunomodulatory potential of the root extract of *A. racemosus*, AgNPs, AuNPs, and Ag-Au alloy NPs is achieved by measuring the cytokine levels in macrophages (IL-1β, IL-6, and TNF-α) and NK cells (IFN-γ) of NK92 and THP1 cells using the solid phase sandwich ELISA technique. The results showed that the root extract of *A. racemosus*, AgNPs, and AuNPs can reduce the pro-inflammatory cytokine levels in the macrophages cells, while Ag-Au alloy NPs can reduce cytokine responses in NK92 cells. Overall, this study shows that the microwave assisted biogenic synthesized bimetallic nanoalloy nanoparticles could be further explored for the development of antibacterial and anti-inflammatory therapies.

## 1. Introduction

Nanotechnology is the branch of technology that deals with the scale of less than 100 nanometers. The chemical and physical properties of materials often change greatly at this scale. It can be used across all the other scientific fields, such as chemistry, biology, physics, engineering, and materials science. Its applications are found in several industries including surface chemistry, medical fields, organic chemistry, semiconductor fabrication and micro fabrication, etc. Nanotechnology helped researchers create new materials with several applications in industries and in the devices with an ultra small size [1]. Nanosized noble metals such as silver and gold have received great attention due to their advanced physicochemical properties and found to have numerous potential applications in the sector of nanoscience including chemistry, biochemistry, biology, and chemical engineering [2,3,4,5].

Among the various metals, silver (Ag) and gold (Au) are noble metals due to their exceptional biomedical and pharmaceutical properties. Silver nanoparticles are the most applied nanostructures, as antibacterial agents in wound healing, pharmaceutical products, and bandages for centuries [6]. Recently, due to their inherent antimicrobial properties, they are also used in other aspects such as packaging, food processing, cosmetic products, textiles, medical instrumental devices, and biosensors [7,8,9,10].

Gold nanoparticles are among the most precious metals. These nanoparticles displayed some unique physical, optical, and catalytic properties. They have been widely applied in many fields, including clinical diagnosis, cancer therapy, and drug delivery systems due to their high stability and low toxicity in biosamples [11,12]. However, bimetallic nanoparticles are of tremendous interest due to their dual effect which can improve their applications. The Ag-Au bimetallic alloy nanoparticles showed potential optical, electronic, catalytic, and biomedical applications [13,14,15]. 

The extensive physicochemical activities of nanoparticles have revolutionized the scientists for eco-compatible approaches in generating nanoparticles, as the use of hazardous chemicals, high energy, and pressure in conventional methods are disastrous to the surrounding environment [16]. The synthesized eco-friendly products possess unique properties that may positively impact their biomedical applications. The huge biodiversity of natural products (plants, microbes, and marine organisms) has tremendous potential which is not fully exploited [17]. Various natural biological resources are yet to be explored for their beneficial use in the preparation of nanoparticles. The recent attention gained by the green synthesis approach is attributed to its simple procedure, minimal use of chemicals, and is biocompatible with the surroundings [18]. Additionally, inherent bioactive molecules, which are easily used as capping and stabilizing agents are major factors that participate in the steady rise of this method adoption. Several studies reported the use of living materials such as plants, bacteria, algae, fungi, and sponge extract for the green synthesis of nanoparticles [19,20,21,22]. In light of the above mentioned useful properties and the ever increasing probability of exposure to monometallic and bimetallic nanoparticles, it is vital to develop an environmental friendly, sustainable synthetic, and cost effective approach for the fabrication of metallic nanoparticles. The use of different natural product extracts, particularly medicinal plants receive much attention due to their low toxicity, non-pathogenicity, and excellent compatibility when compared to different physical and chemical approaches [23]. Previous reports have addressed the biosynthesis of bimetallic nanoparticles using different plant extracts [24,25,26]. 

The biogenic synthesis of nanoparticles (silver, copper oxide, platinum, palladium, etc.) from *Asparagus racemosus* extracts has been reported earlier. These nanoparticles have shown an antibacterial and cytotoxic potential against various bacterial strains and cancer cells, respectively [27,28,29]. The nanoparticles prepared from plant extracts are likely to possess similar biological activities as the plant bears. It depends on whether secondary metabolites responsible for the biological activities of herbal extract have participated in the preparation of the nanoparticles. Nanoparticles derived from plants can enhance the biological activity and bioavailability of the secondary metabolites for the biological properties [30]. *Asparagus racemosus* (*A. racemosus*, Shatavari) is a green edible medical plant belonging to the Liliaceae family and abundantly found in tropical and subtropical regions of Asia, Africa, and Australia. It is frequently used in various Ayurvedic preparations for the treatment of ageing, vigor, to increase immunity, improve longevity, and mental functions. The different parts of this plant find its applications in curing hepatopathy, neurological disorders, dyspepsia, and tumors [31]. Furthermore, various therapeutic properties of roots of *A.racemosus* are well documented in ancient Ayurvedic literature. These therapeutic properties are owing to the numerous pharmacological applications including antioxidant, antimicrobial, anti-inflammtory, and antiseptic effects [31]. The phytochemical investigation revealed that *A.racemosus* contains steroidal saponins as major constituents, along with flavonoids, tannins, and phenolic components [32]. Therefore, the presence of varied phytoconstituents is responsible for the development of novel therapeutic drugs for the cure of various diseases. Since the root extracts of *A. racemosus* have displayed immune modulatory properties [33], it is quite possible that the nanoparticles prepared from this plant may possess immune modulatory effects. Along the same study line, we tried to evaluate the antibacterial and immunomodulatory potentials associated with the roots of *A. racemosus* and biosynthesized metallic Ag, Au, and Ag-Au alloy nanoparticles. 

The key function of the immune system is to protect the body from pathogenic invasion and proliferation of malignant and damaged tissues [34]. The human immune system can be categorized into the innate and adaptive system. The first line of defense, such as macrophage and natural killer cells, is the part of the innate immune system. The main function of these cells is phagocytose pathogens, which is organized by cytokines or interleukins (ILs) [35]. The alteration in ILs secretion is considered as an integral part of the immune modulation system, which helps in new immunotherapies for the cure of cancer, viral, and bacterial infections. The collective action of molecular pathways carefully monitored the immune responses by either suppressing or activating the immune activation. Any disturbance in this chemical balance may result in maximizing the susceptibility to infections, chronic inflammations, and autoimmune diseases in accordance to the overly active or suppressed immune system [36]. These immune disorders could be treated with immunomodulatory drugs, which have ability to stimulate or suppress the immune responses. For instance, drugs that cause the suppression of the immune system could be helpful in the cure of various inflammatory disorders, including rheumatoid arthritis, multiple sclerosis, inflammatory bowel disease, eczema, and psoriasis [37].

Macrophages and NK cells are stimulated by bacterial infections to release pro-inflammatory cytokines (TNF-α, IL-1β, and IFN-γ) to assist the infiltration of immune cells into the diseased tissue [38]. However, certain cases of chronic inflammation (persistent inflammation) can result in undesired complications. In the persistent inflammation, the continual enrollment of innate and adaptive immune cells accelerates the formation of high levels of pro-inflammatory modulators [39]. Patients suffering from chronic inflammation are susceptible to various diseases such as, cancer, diabetes, rheumatoid arthritis, and inflammatory bowel syndrome [40]. Therefore, bacterial infections can be managed by treatment with anti-inflammatory drugs [41]. Various nanomaterials turn up as promising substances for immune modulation application [42]. The analysis of gene expression described that the expression levels of many cytokines such as TNF-α, IL-1β, and IL-6 were influenced in rats after being injected with gold nanoparticles [43]. On the other hand, citrate-gold nanoparticles showed an anti-inflammatory reaction by downregulating the response of cells induced by IL-1β in vitro, as well in vivo [44]. 

In the current study, the root extract of *A. racemosus* was used for the biosynthesis of Ag, Au, and Ag-Au nanoparticles aiming to explore their antibacterial, as well as immunomodulatory activities of the Ag, Au, and Ag-Au bimetallic alloy nanoparticles. The characterization of the biosynthesized nanoparticles of the Ag, Au, and Ag-Au bimetallic alloy was performed by spectroscopic (UV-vis, FTIR, XRD, and EDX) and microscopic (TEM and SEM) analysis. The antibacterial activity was evaluated against different bacterial strains. However, immunomodulatory activities of biosynthesized nanoparticles were investigated against cell culture models of macrophages and NK cells. Moreover, this study compares the antibacterial and immunomodulatory activities Ag, Au, and Ag-Au bimetallic alloy nanoparticles to the *A. racemosus* root extract. 

## 2. Materials and Methods

### 2.1. Botanical Material

Roots of *Asparagus racemosus* Willd (Liliaceae) were purchased from local markets (Jammu Tawi, India) and washed thoroughly with distilled water. Taxonomically, the plant material was authenticated and identified by Prof. Dr. Mohamed Yousef of the Department of Pharmacognosy, College of Pharmacy at King Saud University, Saudi Arabia. A voucher specimen (AR-6402) is deposited in the herbarium of Pharmacognosy Department.

### 2.2. Preparation of Root Biomass 

Roots of *A. racemosus* were dried in an oven at 30 °C and ground to a coarse powder. The powdered material (50 g) was extracted with ethyl acetate in a microwave with 630 W irradiation for 15 min. The extraction process was repeated two more times under the same conditions. The combined extract was filtered through the Whatman No. 1 filter paper and the filtrate obtained was freed from the solvent on a wiped film evaporator at 50 ± 5 °C to get the EtOAc extract residue (8.7 g).

### 2.3. Preparation of Green Synthesis Ag, Au, and Ag-Au Bimetallic Nanoparticles

**Figure 1:**
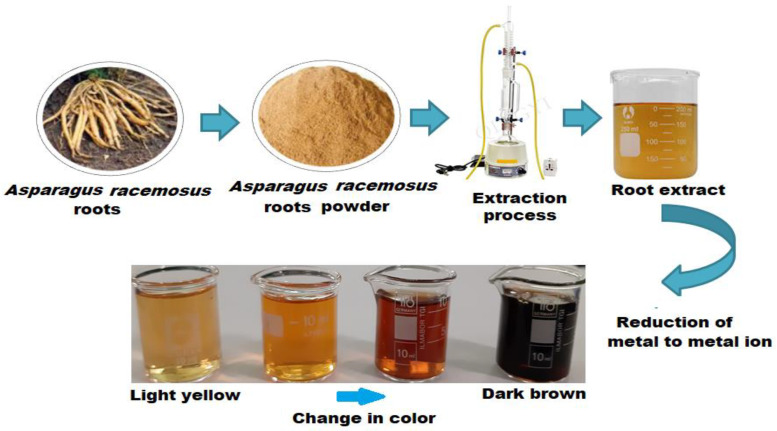
Schematic representation of the green synthesis of Ag-Au alloy nanoparticles by the *Asparargus racemosus* root extract in an ionic liquid medium and sequential color change from light yellow to dark brown.

The biosynthesis of Ag, Au, and Ag-Au bimetallic nanoparticles were synthesized using the *A. racemosus* root extract as previously reported by Lateef et al. [45]. Briefly, 5 mL of the extract was added individually to each reaction vessel containing 50 mL of 1.0 × 10^−3^ mol L^−1^ silver nitrate (AgNO_3_, Sigma-Alsrich, Hamburg, Germany) and chloroauric acid (HAuCl_4_, Sigma-Aldrich, Hamburg, Germany) solution to reduce the Ag^+^ and Au^2+^ ions, while the synthesis of the Ag-Au bimetallic was conducted using the *A. racemosus* root biomass, silver nitrate, and gold chloride solutions. Briefly, 1.0 × 10^−3^ mol L^−1^ aqueous silver nitrate and gold chloride solutions were prepared individually in distilled water. Fifty milliliters of each solution were mixed to produce the Ag-Au salt solution. Ten milliliters of the above prepared *A. racemosus* root biomass were added to 100 mL of the above prepared salt solution [46]. The bioreduction of the reaction mixture was performed in the microwave oven (MARS 6, South San Francisco, CA, USA) by irradiating the mixture for 20 min at a 2.45 GHz frequency and 700 w power and is kept undisturbed at ambient temperature for 24 h in a covered conical flask. The nanoparticles formed were centrifuged for 10 min at 1500 rpm followed by redispersion in 20 mL of distilled water (Figure 1). Further, experiments were performed by adding different volumes (1–10 mL) of plant extracts in a constant Ag-Au salt solution concentration. 

### 2.4. Characterization of Green Synthesis Ag-Au Bimetallic Nanoparticles

The biogenic reductions in Ag^+^ and Au^2+^ ions in the reaction mixture to form bimetallic alloy nanoparticles were visually estimated by the color change and quantitatively monitored by measuring the absorbance spectra of the reaction mixtures using ultra visible-spectrophotometer-2450 (Shimadzu, Kanagawa, Japan). The FTIR of dried-solid Ag-Au nanoparticles were obtained after centrifugation at 1500 rpm for 10 min using KBr pellets (Spectrum BX spectrometer, (PerkinElmer, Waltham, MA, USA). X-ray diffraction (XRD, Shimadzu XRD-6000 diffractometer, Kyoto, Japan) and the selected area diffraction (SAED) were used to determine the size, shape, nature, and alignment of the biosynthesized Ag-Au NPs. Microscopic investigations of prepared Ag-Au NPs were carried out by scanning electron microscopy (SEM, JSM-7610F, JEOL, Akishima, Tokyo, Japan) and transmission electron microscope (TEM, JEM-2100F, JEOL Ltd., Akishima, Tokyo, Japan). The A LYRA3 TESCAN FESEM coupled with energy-dispersive X-ray (EDX, JSM-7610F; JEOL, Akishima, Tokyo, Japan) at 20 kV, was used to determine the elemental composition of the Ag-Au NPs. The surface morphology of synthesized nanoparticles was studied by using coated purified drops of nanoparticles on a glass slide, allowed to dry, and applied to thin films. The zeta potential and hydrodynamic size distribution of the prepared nanoparticles were measured using dynamic light scattering (NanoZS, Malvern Zetasizer Nano series, Worcestershire, UK) in their solutions.

### 2.5. Biomedical Studies for the Ag, Au, and Ag-Au Bimetallic Alloy Nanoparticles

#### 2.5.1. Antibacterial Activities

The antibacterial properties of Ag, Au, and Ag-Au bimetallic nanoparticles were determined by the standard disc diffusion method against *Escherichia coli* (ATCC 25922), *Bacillus subtilis* (ATCC 6633), *Klebsiella pneumonia* (Urine), *Pseudomonas aeruginosa* (ATCC 27853), and *Staphylococcus aureus* (ATCC 25923) bacterial strains [47]. The pre-cultured bacteria (1 × 10^5^ CFU/mL) was swabbed uniformly onto sterile agar plates (Luria Bertani Broth, HiMedia, Mumbai, India). Different concentrations (20, 40, 80 µg mL^−1^) of Ag, Au, and Ag-Au bimetallic nanoparticles were poured onto the plates under sterile condition and incubated at 37 °C for 24 h. After the incubation period, a zone of inhibition was measured and compared with the negative control (water and plant extract) and positive control (Gentamicin and Streptomycin antibiotics).

#### 2.5.2. MIC and MBC Determination of Biosynthesized Nanoparticles

Minimum inhibitory concentrations (MIC) of Ag, Au, and Ag-Au bimetallic alloy nanoparticles against Methicillin-resistant *S. aureus* and *P. aeruginosa* were determined in triplicates by the microdilution method in a Mueller Hinton broth (MHB, HiMedia, Mumbai, India) with streptomycin (25 µg) and gentamycin (25 µg) as a positive control for *S. aureus* and *P. aeuroginosa* strains, respectively. The concentration of the tested samples ranged from 5 to 1920 µg mL^−1^. Briefly, serial dilutions (2-fold) were conducted in 96-well plates where the positive control (broth and microbial cells) was placed in the first column and the negative control (broth and Ag-Au bimetallic alloy nanoparticles) was placed in the last column. An amount of 40 µL of each bacterial suspension was loaded and incubated at 37 °C for 1 day. After 24 h, the absorbance was measured in an ELISA reader (Biotech, Wuxi, China) at 600 nm. For comparison, streptomycin (25 µg) and gentamycin (25 µg) were applied as a positive control, whereas DMSO was used as a negative control. The MBC is described as the minimum concentration of the test component in which no viable bacterial colonies are noticed. The MBC of the biosynthesized AgNPs, AuNPs, and Ag-Au bimetallic nanoparticles were determined by plating aliquots of tubes with no visible growth and the first turbid tube in the MIC series. Treated sample aliquots were added on nutrient agar plates and spread uniformly using a sterile glass L rod and incubated at 37 °C for 12 h.

#### 2.5.3. Morphological Study of *S. aureus* and *P. aeuroginosa*

The effect of Ag-Au alloy on the morphology of both treated and untreated *S. aureus* and *P. aeuroginosa* was evaluated under the scanning electron microscope. The treated bacterial strains were cut into pieces (5–10 mm) and fixed for 1 h in 3% gluteraldehyde in a phosphate buffer saline (pH 7.4) solution followed by another fixation in 2% osmium tetroxide for 1 h. The obtained tissues were dehydrated in ethanol and air dried with carbon dioxide. A silver pain vacuum was used to place the dried tissues on aluminum stubs and were viewed under SEM at 15 kV as an accelerating voltage.

#### 2.5.4. Immunomodulation Activity

The immunomodulatory effects of the Ag, Au, and Ag-Au bimetallic alloy nanoparticles on the cell viability of PMA differentiated human leukemic monocyte cells (THP1) and natural killer cells (NK92) were determined using the WST-1 Cell Proliferation assay as described by the manufacturer (Roche Diagnostics GmbH, Mannheim, Germany). The THP1 cell line was supplied by a Mycobactomics group of King Saud University, Riyadh, Saudi Arabia and maintained in RPMI 1640 supplemented with a Fetal Bovine Serum (FBS, 50%) and penicillin-streptomycin (1%). However, NK92 was procured from the American Type Culture Collection (ATCC). The NK92 cells were maintained in a α-Minimum Essential Medium Eagle (α-MEM) containing Fetal Bovine Serum (12.5%), horse serum (12.5%), recombinant IL-2 (200 UmL^−1^), penicillin-streptomycin (1%), and 2-mercaptoethanol (0.1 mM). The cells were cultured in a humidified incubator under 5% CO_2_ saturation at 37 °C. The cells were treated with different concentrations (25, 50, 100, 200, and 400 μg mL^−1^) of nanoparticles at 37 °C for 24 h. To compensate any interference from the Ag, Au, and Ag-Au bimetallic alloy nanoparticles on the assay, background controls were included as described previously [48]. The 24-well culture plates were seeded with PMA differentiated THP1 and NK92 cells at 2 × 10^5^ cells/100 μL/well density for 1 day. THP1 cells were exposed to a 10 μg mL^−1^ lipopolysaccharide (LPS) cell culture medium for 6 h. On the basis of the WST-1 Cell Proliferation assay results, 200 μg mL^−1^, 30, 25, and 15 nM concentrations of ethyl acetate extract, Ag, Au, and Ag-Au bimetallic alloy nanoparticles, respectively were identified as non-toxic to the cells. After the removal of the LPS medium, the NK92 and LPS activated THP1 cancer cells were subjected to the same treatments for another 18 h. Non-treated cells and LPS activated THP1 for 6 h were taken as negative and positive controls. After 24 h of treatments, the samples were centrifuged for 20 min at 10,000 rpm (Eppendorf, Hamburg, Germany) to be free from cells and nanoparticles. Finally, the supernatants were collected from each well and the concentrations of cytokines were measured as per the manufacturer’s procedure. MaxDiscovery™ ELISA kits (Bioo Scientific, Austin, TX, USA) were used to assess the presence of IL-1β, IL-6, and TNF-α in the supernatant collected from PMA differentiated THP1, whereas IFN-γ concentrations were analyzed from the supernatant of NK92 cells.

## 3. Results and Discussion

### 3.1. Structural Characterization of Nanoparticles

**Figure 2:**
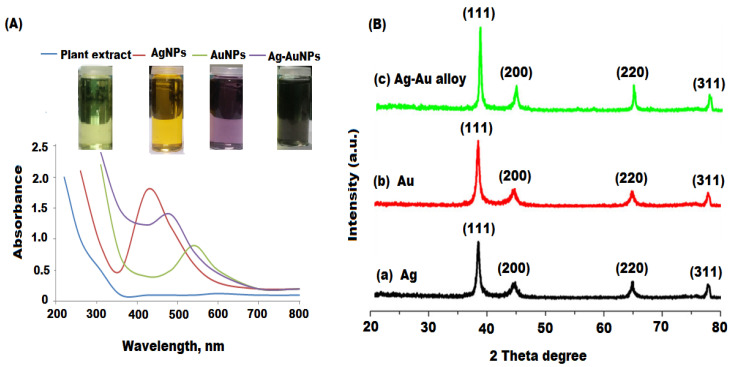
(**A**) UV-vis, (**B**) XRD spectra of plant extract, Ag, Au, and Ag-Au bimetallic alloy nanoparticles.

The biosynthesis of Ag, Au (mono-metallic), and Ag-Au (bimetallic) nanoparticles under the current experimentation could be envisaged by the color change of the reaction mixtures from light yellow to yellow, dark-purple and dark brown, respectively (Figure 1). The reduction conformation of Ag^+^ to Ag^0^, Au^++^ to Au^0^, and Ag-Au NPs was indicated by the color change of the solution from light brown to dark brown. The variation in the brown color indicates the incomplete reduction of less concentration in the plant extract solution, whereas the dark brown color formation at high plant extract concentrations revealed the complete reduction reaction. In the presence of incident photons, Ag and Au nanoparticles displayed the surface plasmon resonance (SPR) band as a result of a collective oscillation of the conduction and free band electrons of the metal [49]. The intensity of the SPR band mainly depends on the nature of the nanoparticles, composition, shape, and matrix used in the synthesis [50]. The synthesis of metal nanoparticles was determined by specific SPR bands and further the formation of Ag, Au, and Ag-Au NPs was confirmed by UV-vis spectroscopy with distinct absorption peaks at 425, 540, and 483 nm, respectively due to the nanoparticles SPR (Figure 2A). Therefore, the root extract of *A. racemosus* served as a reducing and capping agent in the preparation of both mono and bimetallic nanoparticles without using any extra reducing or surfactant agent. Futhermore, the UV-vis spectroscopy is one of the major tools to determine the nature of synthesized Ag, Au, and Ag-Au bimetallic alloy nanoparticles. Previous studies reported that bimetallic alloy nanoparticles usually display two absorption peaks in SPR, whereas a single SPR peak for the alloy type of nanoparticles [51]. The results showed that a single SPR peak appeared between 540 and 425 nm (i.e., Ag and Au nanoparticles SPR peak position) for Ag-Au nanoparticles, indicating the formation of the bimetatllic alloy type. The bimetallic type alloy nanoparticles are formed as a result of the identical lattice constants of Ag and Au, which facilitates their homogeneous distribution within the volume of the particles [52].

The crystalline nature of the synthesized Ag, Au, and Ag-Au bimetallic alloy nanoparticles were determined by XRD. The XRD pattern showed four intense absorption peaks of (111), (200), (220), and (311) parallel to 37.5°, 43.9°, 63.8°, and 76.5°, which confirmed the face centered cubic (fcc) structures for AgNPs and AuNPs with a crystalline nature (Figure 2(Ba,Bb)) [53,54]. However, the results showed that the values of d and 2θ of the Ag-Au bimetallic alloy was very near to each other due to similar lattice constant values (JCPDS: 4-0784 and 4-0783) for Ag and Au metals (Figure 2(Bc)). The XRD pattern of the Ag-Au bimetallic alloy displayed all reflections similar to the Ag and Au monometallic nanoparticles. The calculated mean sizes of the nanoparticles were found to be 20–30 nm with respect to the line width of the maximum intensity peak using the Scherrer equation. 

**Figure 3:**
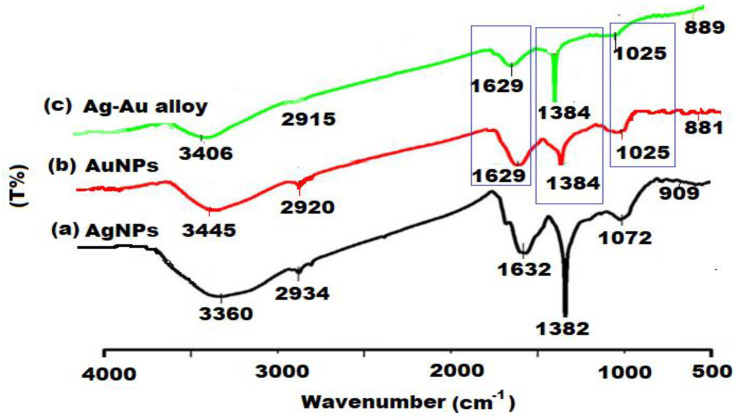
FTIR spectra of Ag, Au, and Ag-Au bimetallic alloy nanoparticles.

The FTIR spectra of biosynthesized Ag, Au, and Ag-Au alloy nanoparticles were performed to identify the nature of possible active constituents which act as a responsible component for the reduction, biocapping, and stabilization of the nanoparticles (Figure 3). The appearance of broad absorption bands in the region 3360 to 3406 cm^−1^ (OH-stretching), 2915 to 2934 cm^−1^ (C–H stretching), 1629 to 1632 cm^−1^ (C–O-stretching), and 881–909 cm^−1^ (spiroketal) indicates the presence of polyphenols, alkanes, amines, and spiroketal saponins, respectively, in the *A. racemosus* root extract [55]. However, the appearance of IR absorption bands from 1382 to 1384 cm^−1^ and 1025 to 1072 cm^−1^ are due to the C–O–H bending of carboxylic acids vibrations and C–O bending vibrations, respectively [56]. On the basis of obtaining FTIR results, the main components present in the ethyl acetate extract of *A. racemosus* are phenolics, steroids, flavonoids, and spiroketal compounds, which were involved in the reduction of metal ions. Moreover, the other chemical constituents, including reducing sugars, carboxylic acids, and amines might also be responsible for the reduction process by donating the free electrons to the metal ions. The protein components (carboxylate, carbonyl, and amine groups) might be responsible for the stabilization of synthesized nanoparticles as they bind to the surface of the nanoparticles and prevent their aggregation. 

**Figure 4:**
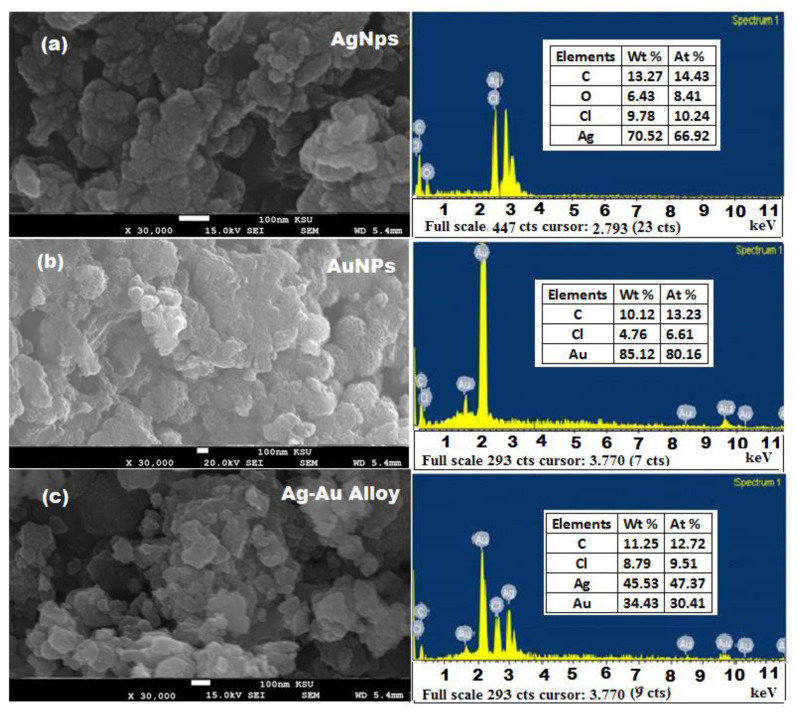
SEM and EDX images of biosynthesized (**a**) Ag, (**b**) Au, and (**c**) Ag-Au alloy nanoparticles (NPs).

**Figure 5:**
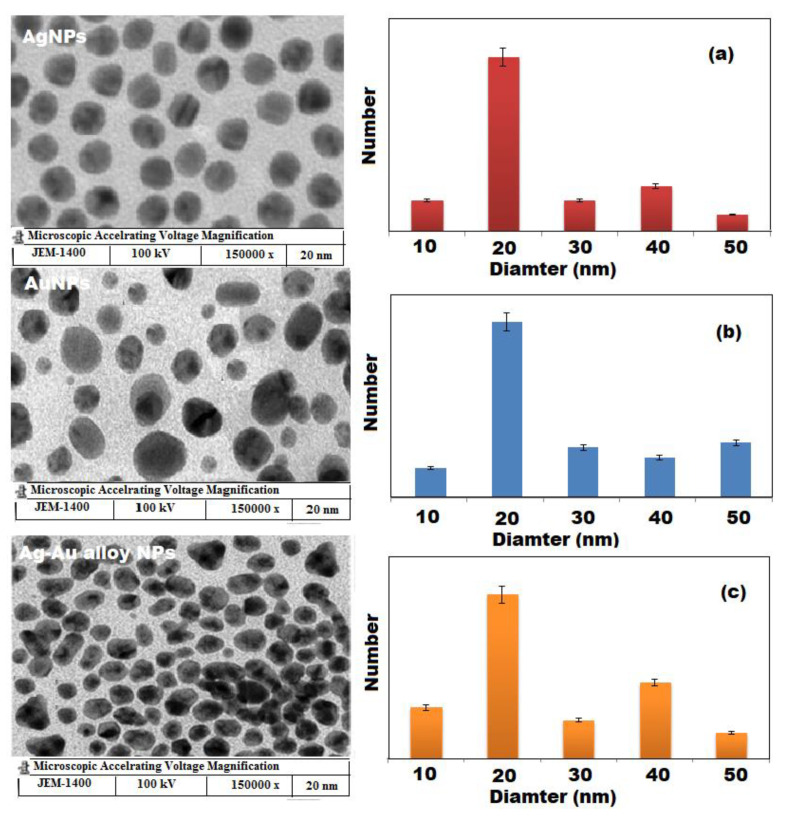
TEM images and particle size distribution of (**a**) Ag, (**b**) Au, and (**c**) Ag-Au alloy NPs.

The SEM images and EDX spectra of biosynthesized Ag, Au, and Ag-Au bimetallic alloy nanoparticles showed that the particles are narrow in size and spherical in shape with a diameter in the range of 10–50 nm (Figure 4). However, some froth was noticed on the surface of these obtained nanoparticles, which could be attributed to the different types of phytochemicals present in the plant extract. Therefore, both FTIR and SEM images confirmed the presence of a huge amount of phytochemicals in the plant extract which can prevent the nanoparticles from agglomeration and helps in the production of stable nanoparticles. There was no other defined morphological difference observed in the preparation Ag, Au, and Ag-Au bimetallic alloy nanoparticles. The EDX analysis of Ag and Au nanoparticles showed that Ag (70.52%) and Au (85.12%) metals are the major elements. However, the elemental composition of Ag-Au bimetallic alloy nanoparticles 45.53:34.43 (Ag:Au), showed the excess of Ag in the Ag-Au bimetallic alloy nanoparticles, indicating that Ag was reduced first in the reaction mixture [57]. The C, O, and Cl were present as minor components due to plant phytochemicals anchored on the surface of the nanomaterials [58]. The morphology and size distribution of synthesized Ag, Au, and Ag-Au bimetallic alloy nanoparticles were investigated by TEM images and particle size distribution histograms (Figure 5). It was observed that all the formed nanoparticles were well dispersed, spherical in shape with a particle size in the range of 10 to 50 nm, and the peak centere was about 20 nm. 

### 3.2. Antibacterial Activity of Ag, Au, Ag-Au Alloy Nanoparticles

**Figure 6:**
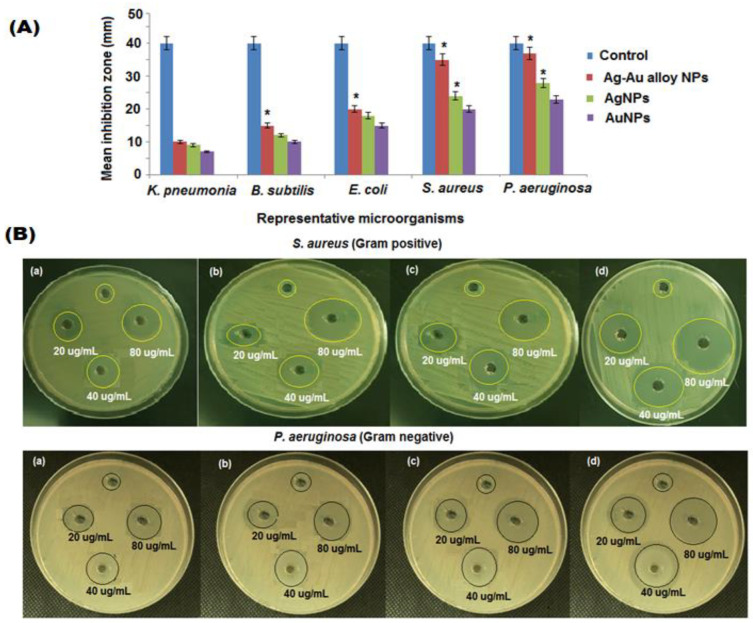
(**A**) Antibacterial activity of plant extract, Ag, Au, and Ag-Au bimetallic alloy nanoparticles against five bacterial strains, (**B**) zone of inhibition (a) control, (b) Ag, (c) Au, (d) Ag-Au bimetallic alloy nanoparticles against *S.aureus* and *P. aeruginosa* bacteria. * *p* < 0.05 compared with untreated group.

The biosynthesized nanoparticles are being extensively utilized in many biomedical applications [59]. The bactericidal effects of nanoparticles are due to killing microorganisms by damaging their cell membrane. The metal and bimetallic nanoparticles, such as the Ag, Au, and Ag-Au bimetallic alloy, have minimum toxicity, pH, and thermal resistance, which provide an excellent antibacterial potential that can be applied in biomedical applications [60]. The biogenic Ag, Au, and Ag-Au bimetallic alloy nanoparticles displayed a promising antibacterial activity against *E. coli*, *B. subtilis*, *K. pneumonia*, *P. aeruginosa,* and *S. aureus* in a dose dependent manner (Figure 6A). As shown in Table 1, the highest antibacterial activity of Ag, Au, and Ag-Au bimetallic alloy nanoparticles were exhibited against *P. aeruginosa* and *S. aureus* (Figure 6B). Three different concentrations (20, 40, and 80 µg mL^−1^) of biosynthesized nanoparticles were treated with *P. aeruginosa* and *S. aureus* bacterial strains. The Ag-Au bimetallic alloy nanoparticles (80 µg mL^−1^) displayed the highest bacterial inhibition against the two chosen bacterial pathogens compared to the single metal Ag, Au nanoparticles, and plant extract. The antibacterial potential of plant extract, Ag, Au, and Ag-Au bimetallic alloy nanoparticles depends on the particle size, shape, surface area, morphology, and polarity of the surface. 

### 3.3. MIC and MBC of Biosythesized Nanoparticles Against S. aureus and P. aeruginosa

**Figure 7:**
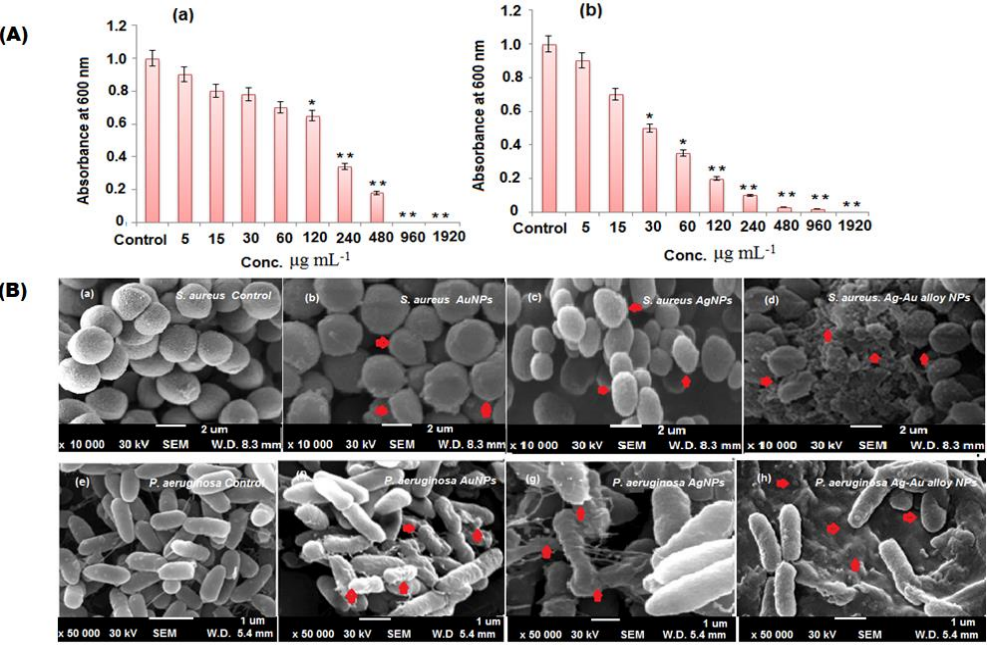
(**A**) Minimum bactericidal concentrations (g mL^−1^) of the Ag-Au alloy nanoparticles against (a) *P*. *aeruoginosa* and (b) *S. aureus.* Results are expressed as the mean SD of triplicate experiments, (**B**) SEM images of untreated and treated with Ag-Au alloy nanoparticles and the altered shape of *P*. *aeruginosa* and *S. aureus.* * *p* < 0.05, ** *p* < 0.01 compared with untreated group.

**Scheme 1:**
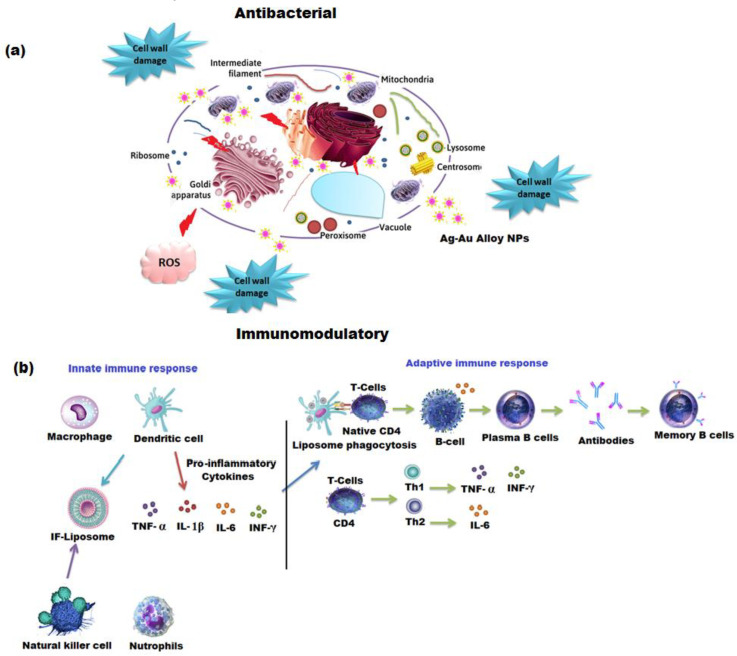
Schematic diagram of the mechanism of (**a**) antibacterial and (**b**) immunomodulatory potentials of Ag-Au alloy NPs.

Bateriostatic and bactericidal concentrations of Ag-Au bimetallic alloy nanoparticles were accessed by the Agar well diffusion procedure. The minimum concentration needed for a visible growth inhibition of *S. aureus* and *P. aeruginosa* was calculated after 1 day incubation at 37 °C. The gradual increase in the concentration (5–1960 μg mL^−1^) of Ag-Au bimetallic alloy nanoparticles showed a significant decrease in viability of bacterial cells (*p* < 0.05). The MIC for *S. aureus* and *P. aeruginosa* was 480 μg mL^−1^ (Figure 7(Aa,Ab)). The MBC for *S. aureus* and *P. aeruginosa* was observed at 480 and 1960 μg mL^−1^ of Ag-Au bimetallic alloy nanoparticles, respectively (Table 2). The antibacterial action of the Ag-Au alloy nanoparticles were enhanced efficiencies compared to the single metal nanoparticles (Ag and Au), which is due to the mixture of Ag and Au ions that can effortlessly participate in attacking the cell membrane of the microorganism [61]. There are two possible mechanisms for the antibacterial activity of the prepared nanoparticles either by photogeneration of reactive oxygen species (superoxide ion, hydroxyl ion, singlet oxygen, and hydrogen peroxide) or by the formation of electrostatic bonds between the bacterial cell membrane and nanoparticles (negative charge of cell membrane interact with positive charge of Ag and Au ions), which leads to the inhibition of microbial growth and induces the death of microganisms. The Ag-Au bimetallic alloy nanoparticles release Ag^+^ and Au^+^ ions, which interact with sulfhydryl or thiol groups (–SH) present on the cell surface and proteins of the cell membrane, resulting in the formation of a steady S-metal group. It leads to the loss of hydrogen ion from the protein molecule and reduces the permeability of the tissue, causing the death of the cell [62]. However, the production of ROS species was enhanced by these nanoparticles through attacking the cell membrane. The ROS generation causes the disruption of DNA, protein, and lipid. The inhibition of enzymes caused by Ag-Au bimetallic alloy nanoparticles were found to be the most significant mechanism, resulting in the damage of the assimilatory food pathway to induce cell demise [63]. The mechanism of Ag-Au bimetallic alloy nanoparticles could be due to the penetration of nanoparticles to the bacterial cell and destroying the microbes as shown in Scheme 1. The XRD and UV results of Ag-Au alloy nanoparticles showed that the small crystalline size and optical band gap energy confirms the enhanced antibacterial activity of Ag-Au bimetallic alloy nanoparticles. Furthermore, SEM and TEM images revealed the spherical shape of Ag-Au alloy nanoparticles nanoparticles, which releases Ag^+^ and Au^+^ ions involved in the inhibition of bacterial growth [64]. The antibacterial activity of plant extract, Ag, Au, and Ag-Au bimetallic alloy nanoparticles against *P. aeruginosa* and *S. aureus* are demonstrated in Scheme 1a. The Ag-Au bimetallic alloy nanoparticles indicated that *P. aeruginosa* (Gram negative) bacteria are more susceptible than *S. aureus* (Gram positive) due to the variation in the composition of their cell wall membrane. *P. aeruginosa* contains a thin peptidoglycan layer in their cell wall compared to *S. aureus* [65]. Therefore, the obtained results confirmed that the Ag-Au bimetallic alloy nanoparticles exhibited an excellent antibacterial potential than Ag and Au nanoparticles. The zone of inhibition of plant extract, Ag, Au, and Ag-Au bimetallic alloy nanoparticles against *P. aeruginosa* and *S. aureus* are shown in Figure 7B.

### 3.4. Morphological study of S. aureus and P. aeruginosa (SEM)

The effect of Ag-Au bimetallic alloy nanoparticles on the surface morphology of *S. aureus* and *P. aeruginosa* was scrutinized under SEM. The treatment of Ag-Au bimetallic alloy nanoparticles has changed the size and shape of selected bacteria due to the nanoparticles coating on the surface bacterial cells as depicted in Figure 7(Bc,Bf). The Ag-Au bimetallic alloy nanoparticles easily penetrate the peptidoglycan membrane of *S. aureus* and *P. Aeruginosa* causing membrane destruction, releasing the contents of the cell, and consequently resulted in cell demise [66]. The untreated bacterial cells were chosen for comparison (Figure 7(Ba,Bd)).

### 3.5. Immunomodulation Activity of Ag, Au, Ag-Au Bimetallic Alloy Nanoparticles

**Figure 8:**
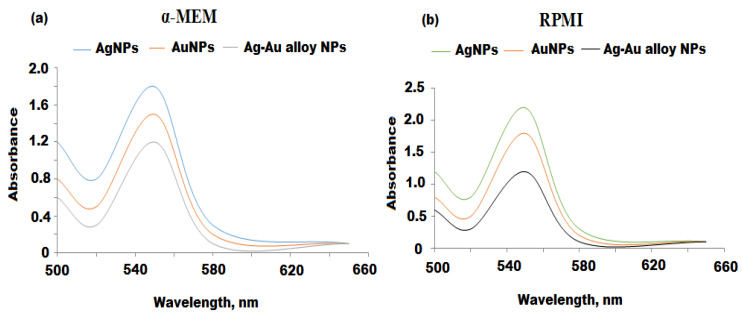
(**a**) UV-vis spectra of Ag, Au, and Ag-Au bimetallic alloy nanoparticles before and after 24 h incubation in the presence of (**a**) α-Minimum Essential Medium Eagle (α-MEM) and (**b**) Royal Park Memorial Institute (RPMI ) cell growth media.

*A. racemosus* has been used in traditional remedies for the cure of various immune-related diseases. Extracts prepared from different parts of *A. racemosus* have shown anti-inflammatory and immunomodulatory properties [33]. It has been suggested that immunomodulatory activities of *A. racemosus* can be attributed to the presence of steroidal saponins as the major constituent [32]. Previous studies have shown that metal-based nanoparticles possess immunomodulatory activities [67]. Herein, the immunomodulatory activities of Ag, Au, and Ag-Au bimetallic alloy nanoparticles were investigated using macrophage and NK cell cultures. The cell culture media were treated with Ag, Au, and Ag-Au alloy nanoparticles in vitro immunomodulatory assays. The cell culture medium is composed of proteins, amino acids, peptides, carbohydrates, minerals, and buffering agents. If any constituents of this medium react with the synthesized Ag, Au, and Ag-Au bimetallic alloy nanoparticles, the biophysical properties of these nanoparticles will be altered. This can also cause the aggregation of Ag, Au, and Ag-Au bimetallic alloy nanoparticles. Therefore, the stability of Ag, Au, and Ag-Au bimetallic alloy nanoparticles were tested on a α-MEM and RPMI cell culture media. This process was conducted by placing Ag, Au, and Ag-Au bimetallic alloy nanoparticles in a α-MEM and RPMI medium for 24 h and measured spectrophotometerically to determine the change of the UV-vis spectra of the A Ag, Au, and Ag-Au bimetallic alloy nanoparticles, as previously reported [68]. Any observed changes such as red shift or broadening of the absorption peak in the UV-vis spectrum of Ag, Au, and Ag-Au bimetallic alloy nanoparticles, will indicate the increase or decrease in size of these nanoparticles or their aggregation [69]. Figure 8a,b showed that the α-MEM and RPMI media did not affect the UV-vis spectrum of investigated (Ag, Au, and Ag-Au bimetallic alloy nanoparticles), suggesting their stability in these media.

The immunomodulatory effects of the *A. racemosus* root extract, Ag, Au, and Ag-Au bimetallic alloy nanoparticles were assessed against differentiated THP1 and NK92 cell lines. Monocytes and NK cells are the integral part of the immune system, which produce various ILs that affect the functions of other cells and control the immune response to different infections [70]. 

**Figure 9:**
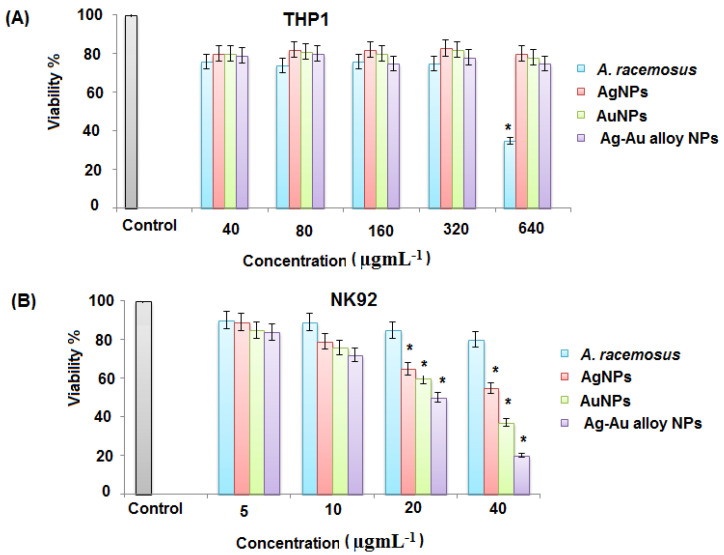
The effect of *A. racemosus,* Ag, Au, and Ag-Au bimetallic alloy nanoparticles on the viability of (**A**) Phorbol 12-myristate 13-acetate (PMA ) differentiated human leukemic monocyte cells (THP1) and (**B**) natural killer cells (NK92) as determined by the water soluble tetrazolium salt (WST-1 ) assay. * Statistically significant (*p* ˂ 0.05) compared to the negative control.

**Figure 10:**
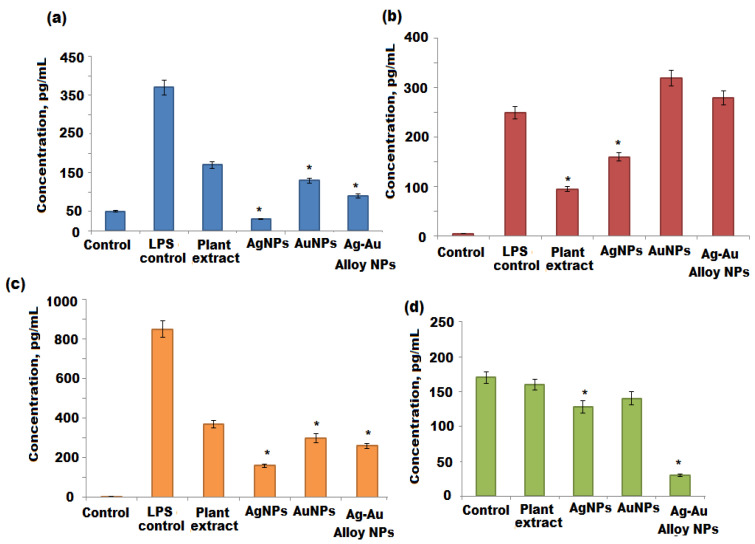
Quantification of cytokine release from THP1 and NK92 cells following treatment with *A. racemosus,* Ag, Au, and Ag-Au bimetallic alloy nanoparticles. (**a**–**c**) THP1 cells were stimulated with lipopolysaccharide (LPS) for 6 h. The LPS containing medium was then replaced by the respective treatments and the cells were incubated for another 18 h, after which the cytokine production (IL-1β, IL-6, and TNF-α) was quantified by ELISA. (**d**) NK92 cells were exposed to the respective treatments for 24 h, after which the IFN-γ production was quantified by ELISA. Statistical significance (*p* < 0.05) compared to the the negative control. * Statistical significance (*p* < 0.05) compared to the 6 h treatment with LPS (LPS control).

The WST-1 viability assay was used to determine the toxicity of the *A. racemosus* root extract, Ag, Au, and Ag-Au bimetallic alloy nanoparticles against differentiated THP1 and NK92 cells. The concentrations of biosynthesized Ag, Au, and Ag-Au bimetallic alloy nanoparticles ranged from 2 to 40 nM, whereas the concentration of *A. racemosus* root extract ranged from 40 to 520 μg mL^−1^. The results showed that the viability of differentiated THP1 cells was less affected by Ag, Au, and Ag-Au bimetallic alloy nanoparticles, while the viability of NK92 cells was significantly affected by Ag, Au, and Ag-Au bimetallic alloy nanoparticles at the highest dose of 40 nM. The viability of differentiated THP1 cells was reduced by 15%, 20%, 25% when treated with Ag, Au, and Ag-Au bimetallic alloy nanoparticles, respectively. The NK92 cell viability was decreased by 30% and 38% when treated with Ag and Au nanoparticles, while the viability of NK92 cells was decreased by 53% when treated with Ag-Au bimetallic alloy nanoparticles at a dose of 40 nM (Figure 9A). However, the NK92 cells were significantly affected by the *A. racemosus* extract at the highest dose, 520 μg mL^−1^ (Figure 9B). Based on these results, 20 nM of each synthesized nanoparticle (Ag, Au, and Ag-Au bimetallic alloy nanoparticles) and 260 μg mL^−1^ of *A. racemosus* root extract was used to evaluate their immunomodulatory potential towards differentiated THP1 and NK92 cells. PMA was used to differentiate THP1 cells into macrophage-like cells before treating them with the *A. racemosus* root extract, Ag, Au, and Ag-Au bimetallic alloy nanoparticles. A bacterial endotoxin LPS was used to treat the differentiated THP1 cells after the exposure to PMA. This bacterium helps trigger the pro-inflammatory responses in the THP1 macrophage-like cells and activates the secretion of pro-inflammatory cytokines such as TNF-α, IL-1β, IL-6, and IFN-γ [71]. TNF-α is among the early released cytokines from the macrophages after the infections and is known as “master regulator” of the pro-inflammatory cytokines [72]. IL-1β plays an important role in the activation of antigen-presenting cells, which in turn results in the formation of aggressive adaptive immune cells towards infections. IL-6 is considered another important immune system regulator, which serves in both pro- and anti-inflammatory actions. The pleiotropic action of IL-6 is based on the activation of either the pro- or anti-inflammatory signaling pathway [73]. A diagnostic feature for early infection of bacteria can be determined by the increased levels of IL-6. The LPS treated differentiated THP1 cells for 6 h exhibited a remarkable increase in TNF-α, IL-1β, and IL-6 levels compared to the untreated THP1 cells (negative control) (Figure 10a–c). Stimulated differentiated THP1 cells after the treatment with LPS for 6 h were then treated with the *A. racemosus* root extract, Ag, Au, and Ag-Au bimetallic alloy nanoparticles for 18 h. The results showed a remarkable reduction in TNF-α and IL-1β levels in comparison to the LPS treated differentiated THP1 cells (LPS control). It was observed that the *A. racemosus* root extract, Ag, Au, and Ag-Au bimetallic alloy nanoparticles were non-toxic to the cells at these doses (40, 80, 160, and 320 µg mL^−1^), indicating that these treatments showed a significant anti-inflammatory response in macrophage-like THP1 cells. The *A. racemosus* root extract also showed the IL-6 response by decreasing the cells in differentiated THP1 cells. However, the treatments with Ag, Au, and Ag-Au bimetallic alloy nanoparticles did not show any IL-6 responses in differentiated THP1 cells. A moderate elevation in IL-6 levels was noticed in differentiated THP1 treated cells Ag, Au, and Ag-Au bimetallic alloy nanoparticles compared to cells treated with LPS for 6 h. Therefore, a higher dose or longer treatments of Ag, Au, and Ag-Au bimetallic alloy nanoparticles could lead to a significant increase of the IL-6 response. The influence of the synthesized (Ag, Au, and Ag-Au bimetallic alloy) nanoparticles and *A. racemosus* extract on the cytokine responses in the NK92 cells, in particular IFN-γ cytokine, were evaluated. NK cells, mainly produce IFN-γ cytokine as a major effector in response to bacterial and viral infections [74]. The treatment of NK92 cells with the *A. racemosus* extract resulted in less production of IFN-γ levels. The production level of IFN-γ was considerably reduced with Ag and Au nanoparticles. However, a significant reduction in IFN-γ production was observed in Ag-Au alloy nanoparticles treated NK92 cells (Figure 10d) compared to the untreated cells (negative control). The IFN-γ levels in treated Ag-Au bimetallic alloy nanoparticles were 15 ± 4 pg mL^−1^, compared to 79 ± 12 pg mL^−1^ for the negative control. Previous studies revealed that IFN-γ increases the production of IL-1β and TNF-α pro-inflammatory cytokines [75]. In this study, the Ag-Au bimetallic alloy nanoparticles showed strong effects on NK92 cells, which can be perceived as an anti-inflammatory response. The obtained response can be described as the synergistic effect of Ag^+^ and Au^+^ ions with plant extract, which might have converted the bimetallic alloy nanoparticles to a more active form compared to the individual Ag and Au metal nanoparticles. The FTIR spectrum of Ag-Au bimetallic alloy nanoparticles also suggested a noticeable modification in the chemistry of prepared Ag-Au bimetallic alloy nanoparticles (Scheme 1b). In the present study, the moderate elevation in IFN-γ production levels in the negative control confirms that the IL-2 used in culturing the NK92 cells also caused the elevation of IFN-γ production [76,77]. 

Inflammation is an important mechanism of the innate immune system to control viral and bacterial infections [78]. During infections, the increased pro-inflammatory cytokine production triggers a cascade of events that cause the intrusion of infected tissues by innate immune cells. However, adverse health effects may be caused by the overproduction of pro-inflammatory cytokine in response to infections. The release of lytic enzyme or oxidative stress can also cause the irreversible damage to the inflamed tissues [79]. The elevation in levels of IL-1β is responsible for various inflammatory disorders such as psoriasis and rheumatoid arthritis [80]. However, the decrease in TNF-α levels is considered a key therapy for rheumatoid arthritis [81]. Furthermore, many inflammatory disorders including psoriasis, rheumatoid arthritis, inflammatory bowel disease, eczema, and multiple sclerosis can benefit from anti-inflammatory medications. The outcome of this study revealed the anti-inflammatory responses of *A. racemosus* root extract, Ag, Au, and Ag-Au bimetallic alloy nanoparticles in THP1 differentiated cells and NK92 cells. 

## 4. Conclusions

The biogenic green synthesis of single metallic Ag, Au, and Ag-Au bimetallic alloy nanoparticles prepared by the microwave assisted method using the ethyl acetate root extract of *A. racemosus* was described. The prepared nanoparticles (metallic and bimetallic) characterized by several analytical techniques including UV-vis, FTIR, XRD, SEM with the EDX and TEM analysis confirmed that synthesized metallic and bimetallic nanoparticles face a centered cubic structure. The average size of crystalline Ag, Au, and Ag-Au bimetallic alloy nanoparticles were around 10–50 nm. The SEM and TEM micrographs of Ag-Au alloy nanoparticles are spherical in shape with a uniform agglomeration. The EDX spectra strongly represented that silver and gold metals exist in the Ag-Au bimetallic alloy nanoparticles matrix. The FTIR spectra confirmed the functional group present in the plant extract, as well as in the prepared nanoparticles. Moreover, this study summarized that the biogenic synthesized Ag-Au bimetallic alloy nanoparticles exerted excellent antibacterial and immunomodulatory potentials compared to the individual (Ag and Au) nanoparticles and plant extract. The outcome of the study supports the utilization of this traditional plant for the treatment of inflammation. The Ag, Au, and Ag-Au bimetallic alloy nanoparticles reduced the secretion of pro-inflammatory cytokines in THP1 cells (PMA differentiated). The Ag-Au bimetallic alloy nanoparticles significantly reduced the secretion of IFN- γ and affected the response of cytokines in NK92. These nanoparticles can be further exploited for the treatment of anti-inflammatory effects.

## Figures and Tables

**Table 1:** Antibacterial activity of the *A.racemosus* extract, Ag, Au, and Ag-Au alloy NPs against Gram positive and Gram negative bacteria.

S.No.	Samples	Antibacterial Activity			
		*S. aureus*		*P. aeruginosa*	
		Concentration (µg mL^−1^)	Zone of Inhibition (mm)	Concentration (µg mL^−1^)	Zone of Inhibition (mm)
1	Plant extract	20	15	20	17
		40	19	40	21
		80	20	80	22
2	AgNPs	20	18	20	21
		40	22	40	24
		80	24	80	28
3	AuNPs	20	17	20	19
		40	20	40	24
		80	23	80	26
4	Ag-Au alloy NPs	20	22	20	25
		40	26	40	29
		80	30	80	33

**Table 2:** Minimum bacetericidal activity of Ag-Au alloy nanoparticles against *P*. *aeruginosa* and *S. aureus*.

Sample	CFU mL^−1^	
	*P. aeruoginosa*	*S. aureus*
Control	TNTC	TNTC
5	TNTC	TNTC
15	TNTC	TNTC
30	TNTC	TNTC
60	TNTC	TNTC
120	TNTC	TNTC
240	5 × 10^4^	3 × 10^2^
480	1 × 10^2^	148
960	NIL	5
1920	NIL	1

## Data Availability

The data used to support the findings of this study are included within the article.
